# The Apennines as a cryptic Pleistocene refugium of the bark beetle *Pityogenes chalcographus* (Coleoptera: Curculionidae)

**DOI:** 10.1093/biolinnean/blz012

**Published:** 2019-04-05

**Authors:** Martin Schebeck, Hannes Schuler, Birgit Einramhof, Dimitrios N. Avtzis, Eddy J. Dowle, Massimo Faccoli, Andrea Battisti, Gregory J. Ragland, Christian Stauffer, Coralie Bertheau

**Affiliations:** 1 Department of Forest and Soil Sciences, University of Natural Resources and Life Sciences Vienna, BOKU, Vienna, Austria; 2 Faculty of Science and Technology, Free University of Bozen-Bolzano, Bozen-Bolzano, Italy; 3 Forest Research Institute, Hellenic Agricultural Organization Demeter, Thessaloniki, Greece; 4 Department of Integrative Biology, University of Colorado Denver, Denver, CO, USA; 5 Department of Anatomy, University of Otago, Dunedin, New Zealand; 6 Department of Agronomy, Food, Natural Resources, Animals and Environment (DAFNAE), University of Padua, Padua, Italy; 7 Laboratoire Chrono-Environnement, Université de Bourgogne Franche-Comté, Pôle Universitaire du Pays de Montbéliard, Montbéliard, France

**Keywords:** Apennines, biogeography, *COI*, ddRADSeq, ice ages, phylogeography, Pleistocene, population genetics, Scolytinae, *Picea abies*

## Abstract

The Apennine Mountains in Italy are an important biogeographical region and of particular interest in phylogeographical research, because they have been a refugium during Pleistocene glaciation events for numerous European species. We performed a genetic study on the Eurasian bark beetle *Pityogenes chalcographus* (Linnaeus, 1760), focusing on two Apennine (Italian) and two Central European (Austrian) locations to assess the influence of the Apennines in the evolutionary history of the beetle, particularly during the Pleistocene. We analysed a part of the mitochondrial *COI* gene and a set of 5470 informative genome-wide markers to understand its biogeography. We found 75 distinct mitochondrial haplotypes, which are structured in three main clades. In general, the Apennine locations harbour a higher number of mitochondrial clades than Central European sites, with one specific clade exclusively detected in the Apennines. Analysis of our genome-wide, multi-locus dataset reveals a clustering of *P. chalcographus* by geography, with Italian individuals clearly separated from Austrian samples. Our data highlight the significance of the Apennines for the genetic diversity of *P. chalcographus* and support the hypothesis that this area was an important refugium during unfavourable conditions in the Pleistocene. We discuss additional life-history traits and processes that shaped the evolution of this widespread beetle.

## Introduction

The Apennine Peninsula is an interesting biogeographical region and a significant source of diversity for numerous European animal and plant species (Bartolini *et al.*, 2014; Dapporto *et al.*, 2014; Bogdanowicz *et al.*, 2015; Piotti *et al.*, 2017; Vitecek *et al.*, 2017). This area in the Mediterranean region is a biodiversity hotspot and is important for understanding the phylogeography of numerous species (Blondel *et al.*, 2000; Hewitt, 2000; Schmitt, 2007). In particular, the Apennines were a refugium, where many vertebrates, invertebrates and plants were able to survive unfavourable environmental conditions, such as glacial periods during the Pleistocene (Hewitt, 1996). Range contractions and expansions, limited gene flow and secondary contact during glacial–interglacial cycling explain much of the present genetic structure of various European organisms (Hewitt, 2000), and fine-scale analyses using molecular tools can help us to understand their evolutionary history (Beichman *et al.*, 2018).

Bark beetles (Coleoptera: Curculionidae: Scolytinae) are a diverse and species-rich insect group (Hulcr *et al.*, 2015). They spend the most part of their life cycles in various plant tissues and play important roles in many forest ecosystems (Raffa *et al.*, 2015). Bark beetles are characterized by their long-standing and close association with plants, resulting in mutual adaptions to each other and overlapping distribution areas of a beetle and its main host. Climatic fluctuations during the Pleistocene with parallel range changes of insect and plant were major evolutionary drivers in various scolytine species (Stauffer *et al.*, 1999; Cognato *et al.*, 2003; Mock *et al.*, 2007; Avtzis *et al.*, 2008; Bertheau *et al.*, 2013; Schebeck *et al.*, 2018a).

*Pityogenes chalcographus* (Linnaeus, 1760) (Coleoptera: Curculionidae) is a widespread bark beetle in Eurasia. It infests several conifer species, with Norway spruce, *Picea abies* [L.] Karst., as preferred host plant, reflected by geographical range overlaps of insect and host (Pfeffer, 1995) and by highest preference–performance patterns compared with other Pinaceae species (Bertheau *et al.*, 2009). The phylogeographical signal of *P. chalcographus* was considerably shaped by range contractions and expansions during the Pleistocene and co-evolutionary dependencies on Norway spruce (Avtzis *et al.*, 2008; Bertheau *et al.*, 2013). Main glacial refugia of *P. chalcographus* where hypothesized for the Carpathian Mountains, the Russian plain and the Italian–Dinaric region, including the Apennine Mountains (Avtzis *et al.*, 2008; Bertheau *et al.*, 2013; Schebeck *et al.*, 2018a). Information on the location of putative refugia was derived from molecular markers of *P. chalcographus* (Avtzis *et al.*, 2008; Bertheau *et al.*, 2013; Schebeck *et al.*, 2018a) combined with genetic and pollen data of Norway spruce (Schmidt-Vogt, 1977; Tollefsrud *et al.*, 2008). One particular Pleistocene refugium of Norway spruce was located in the Apennines, and this region represents the southernmost part of its present range on the Italian Peninsula (Schmidt-Vogt, 1977; Giannini *et al.*, 1991). Thus, *P. chalcographus* probably shared this refugium with its main host; however, a fine-scale genetic analysis of this region has been lacking so far.

Mitochondrial genetic analyses revealed that European *P. chalcographus* is structured in three main *COI* clades (hereafter called PcI, PcII and PcIII), with clade PcIII being highly diverse, reflected by four sub-clades (PcIIIa–PcIIId) (Avtzis *et al.*, 2008; Bertheau *et al.*, 2013). The geographical distribution of these mitochondrial clades shows a general pattern: PcI is mainly found in Northern European populations and PcIIIa predominately in Central Europe, whereas southern European populations show a relatively high diversity of mitochondrial haplotypes. Furthermore, the analysis of genome-wide single nucleotide polymorphism (SNP) markers revealed that the genetic structure of Eurasian *P. chalcographus* largely follows a geographical pattern, and it is characterized by high levels of admixture and low levels of differentiation among locations (Schebeck *et al.*, 2018a). The biogeography of this bark beetle was hypothesized to be a result of climate-driven range contractions to multiple refugia during the last ice ages and subsequent postglacial recolonization (Avtzis *et al.*, 2008; Bertheau *et al.*, 2013; Schebeck *et al.*, 2018a).

These studies, however, lack a thorough and comprehensive fine-scale analysis of Apennine *P. chalcographus*, and knowledge on the influence of this region on the genetic structure of Central European populations is scarce. Based on previous data, we hypothesize that within the Italian–Dinaric region the Apennine Mountains were a Pleistocene glacial refugium, shared with Norway spruce, that considerably affected the genetic structure of Central European beetles. Furthermore, study of molecular markers can detect past evolutionary signatures, reflected by the species’ present phylogeographical signal. Therefore, we performed a biogeographical study focusing on individuals from the Apennines and compared them with beetles from adjacent locations from the Northern Alps in Central Europe. Comprehensive analysis of a part of the mitochondrial *COI* gene and a set of genome-wide SNP markers will reveal new and detailed insights into the genetic structure of European *P. chalcographus* and provide a better understanding of its evolutionary history.

## Material and Methods

### Sample collection and DNA extraction

Adult *P. chalcographus* individuals were collected from breeding systems of standing trees or felled logs of Norway spruce from two Apennine [i.e. Italy/Abetone (ITAB) and Italy/Pavullo (ITPA)] and two Central European [i.e. Austria/Prinzersdorf (ATPR) and Austria/Rothwald (ATRO)] locations (Table 1). Only one beetle per breeding system was sampled to avoid the analysis of siblings. Beetles were stored in absolute ethanol at −20 °C, and DNA was extracted using the GenElute Mammalian Genomic DNA miniprep kit (Sigma-Aldrich, St Louis, MO, USA) according to the manufacturer’s protocol.

### Mitochondrial DNA

In total, 190 individuals (ITAB, *N* = 48; ITPA, *N* = 48; ATPR, *N* = 47; ATRO, *N* = 47) were analysed (Table 1). A part of the mitochondrial *COI* gene was PCR amplified using the forward primer PcCOIF (Bertheau *et al.*, 2013) and the reverse primer UEA10 (Lunt *et al.*, 1996). The PCRs were carried out in a total volume of 25 µL, using 2 mM MgCl_2_, 100 µM dNTPs, 0.5 µM forward and reverse primer each, 1 U Taq polymerase (Fermentas, Lithuania, Vilnius) and ~50 ng template DNA. The PCR conditions were 94 °C for 3 min, followed by 33 cycles of 94 °C for 30 s, 48 °C for 60 s and 68 °C for 90 s, followed by a final extension step at 68 °C for 10 min.

The PCR products were purified using the GenElute PCR Clean-Up kit (Sigma-Aldrich) and sequenced by a commercial provider (Cancer Research Center Sequencing Facility, University of Chicago, Chicago, IL, USA). Sequences were edited in *Bioedit* v.7.0.5 (Hall, 1999) and aligned with *Clustal W* (Thompson *et al.*, 1994). To avoid false positives, haplotypes found in only one individual were confirmed by an additional, independent PCR run.

Population genetic analyses of *COI* data were performed in MEGA6 (Tamura *et al.*, 2013); that is, number of haplotypes, number of informative sites, nucleotide diversity and pairwise genetic distances (Kimura two-parameter, i.e. K2P). The phylogenetic relationship among haplotypes was studied by using a maximum-likelihood (ML) approach (1000 bootstrap replicates, K2P distances).

### Genomic DNA

A total of 30 individuals from the two Italian (ITAB, *N* = 9; ITPA, *N* = 12) and from one Austrian site (ATRO, *N* = 9) were studied (Table 1). To obtain high-resolution insight into the population structure of *P. chalcographus*, we applied double digest restriction site-associated DNA sequencing (ddRADSeq) (Parchman *et al.*, 2012), as used by Egan *et al.* (2015), and analysed a set of 5470 informative loci (Schebeck *et al.*, 2018a; Supplementary Information SI 1). In brief, 200 ng genomic DNA per individual was restriction digested using the enzymes EcoRI and MseI. EcoRI adapters with an individual barcode, as used by Egan *et al.* (2015), and MseI adapters were ligated to these digested fragments and amplified (30 PCR cycles). Afterwards, samples were pooled and purified with magnetic beads (Agencourt AMPupre XP; Beckman Coulter, Indianapolis, IN, USA), followed by a size selection for 400–500 bp fragments using a BluePippin System (Automated DNA Size Selection System; Sage Science, Beverly, MA, USA). Quality control was done on a Bioanalyzer 2100 (Agilent, Santa Clara, CA, USA) and a Qubit 2.0 Fluorometer (Invitrogen, Karlsruhe, Germany). Libraries were sequenced for 100 bp reads on a single Illumina HiSeq2000 lane (BGI Americas, Cambridge, MA, USA).

After sequencing, raw reads were de-multiplexed using custom scripts available from Assour *et al.* (2015) and Dowle *et al.* (2017). A de novo ‘pseudo-reference genome’ was built in *Stacks* v.1.35 by applying *ustacks* (Catchen *et al.*, 2011, 2013) as used by Schebeck *et al.* (2018a). Reads were aligned using *BWA-MEM* v.0.7.12 (Li & Durbin, 2009), and SNPs were called in *GATK* v.3.3.0, applying the UnifiedGenotyper (McKenna *et al.*, 2010). The SNPs were filtered to remove those with a base and mapping quality phred score < 20, a total depth per locus across individuals less than ten, and minor allele frequency < 0.05. Furthermore, SNPs were discarded when sites were over-assembled, that is, when the null hypothesis of a χ^2^ test that each allele in a heterozygote individual has a representation of 0.5 is rejected at a *P*-value of < 0.05. Loci passing these filters were subsequently analysed in ANGSD v.0.913 (Korneliussen *et al.*, 2014), and genotypes were called by applying the *GATK* model. Individuals were used for subsequent analyses only when the missing-data rate was < 30%.

To assess the genetic population structure of *P. chalcographus*, we calculated a discriminant analysis of principal components (DAPC) (Jombart *et al.*, 2010) as implemented in *adegenet* v.2.0.1 (Jombart, 2008; Jombart & Ahmed, 2011) from called genotypes. To assess the optimal number of principal components, in order to find optimal discrimination of clusters and prevent over-fitting, the α-score was used.

Furthermore, pairwise *F*_ST_ values among geographical sites were calculated from called genotypes via *adegenet* (Jombart, 2008; Jombart & Ahmed, 2011).

## Results

### *COI* analyses

**Figure 1:**
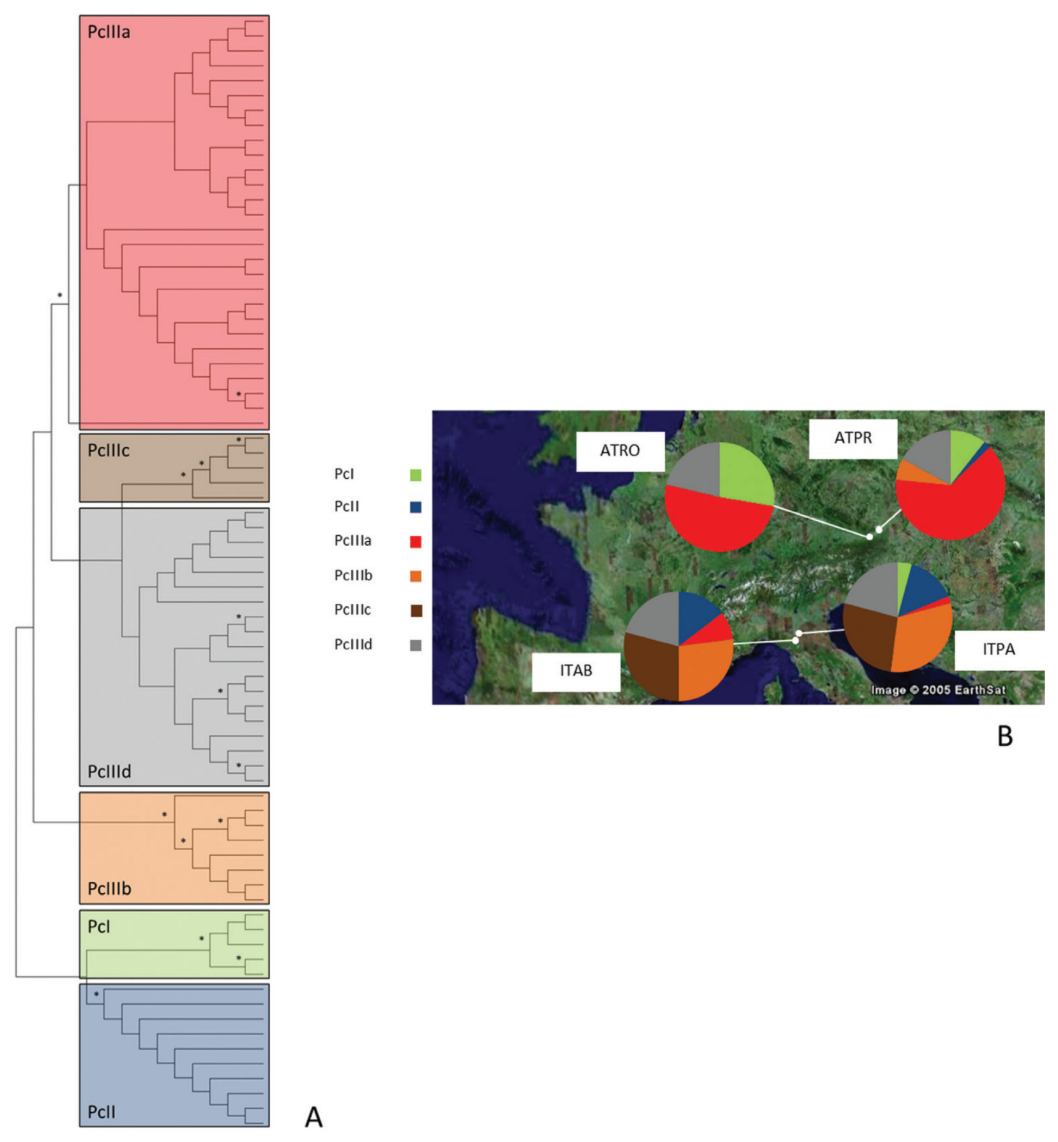
A, relationship among mitochondrial *COI* haplotypes of *Pityogenes chalcographus* using a maximum-likelihood approach (1000 bootstrap replicates, Kimura two-parameter). *Bootstrap value > 60%. B, proportions of mitochondrial *COI* clades in four locations (ATPR, Austria/Prinzersdorf; ATRO, Austria/Rothwald; ITAB, Italy/Abetone; ITPA, Italy/Pavullo). Clade terminology after Avtzis *et al.* (2008) and Bertheau *et al.* (2013).

Analysing an 869 bp fragment of the mitochondrial *COI* gene resulted in 75 distinct haplotypes with 80 polymorphic sites, of which 46 were parsimony informative (GenBank accession numbers: MK423763–MK423837). Thirty-four haplotypes were found only once. By applying an ML approach, the haplotypes were found to be structured in six clades/subclades [Fig. 1A; Supporting Information, Table S1; clade terminology follows Avtzis *et al.* (2008) and Bertheau *et al.* (2013)]. Clade PcI comprises five haplotypes, clade PcII ten haplotypes, and clade PcIII consists in total of 60 haplotypes and is structured in four subclades (PcIIIa–PcIIId). Apart from PcIIId, all clades are supported by bootstrap values > 60% (asterisks in Fig. 1A). The maximal genetic distance among haplotypes was 2.3% (between haplotypes of PcI and PcIIId; mean ± SE = 1.1 ± 0.2%; Supporting Information, Table S2), and the average distance among clades was 1.4 ± 0.08% (Table 2). A mean diversity of 0.007 was calculated.

The distribution of mitochondrial clades shows geographical differences (Fig. 1B; Supporting Information, Table S1). Individuals from the Central European location ATRO were assigned to three mitochondrial clades: 51% belonged to PcIIIa, 28% to PcI, and 21% to PcIIId. In the other Central European site, ATPR, haplotypes of five mitochondrial clades were detected. Again, the majority belonged to PcIIIa (64%), 17% to PcIIId and 11% to PcI. A smaller number of samples were assigned to PcII (2%) and PcIIIb (6%).

In contrast, haplotypes of PcI and PcIIIa were rarely detected in both Apennine locations (ITAB: PcI 0%, PcIIIa 8%; and ITPA: PcI 4%, PcIIIa 2%; Fig. 1B; Supporting Information, Table S1). Most individuals were assigned to PcIIIb (ITAB 27%, ITPA 31%) and PcIIIc (ITAB 29%, ITPA 27%). In both Apennine locations, 21% of haplotypes belonged to PcIIId and 15% to PcII. It is noteworthy that clade PcIIIc was found exclusively in the Apennine region.

### ddRADSeq analyses

**Figure 2:**
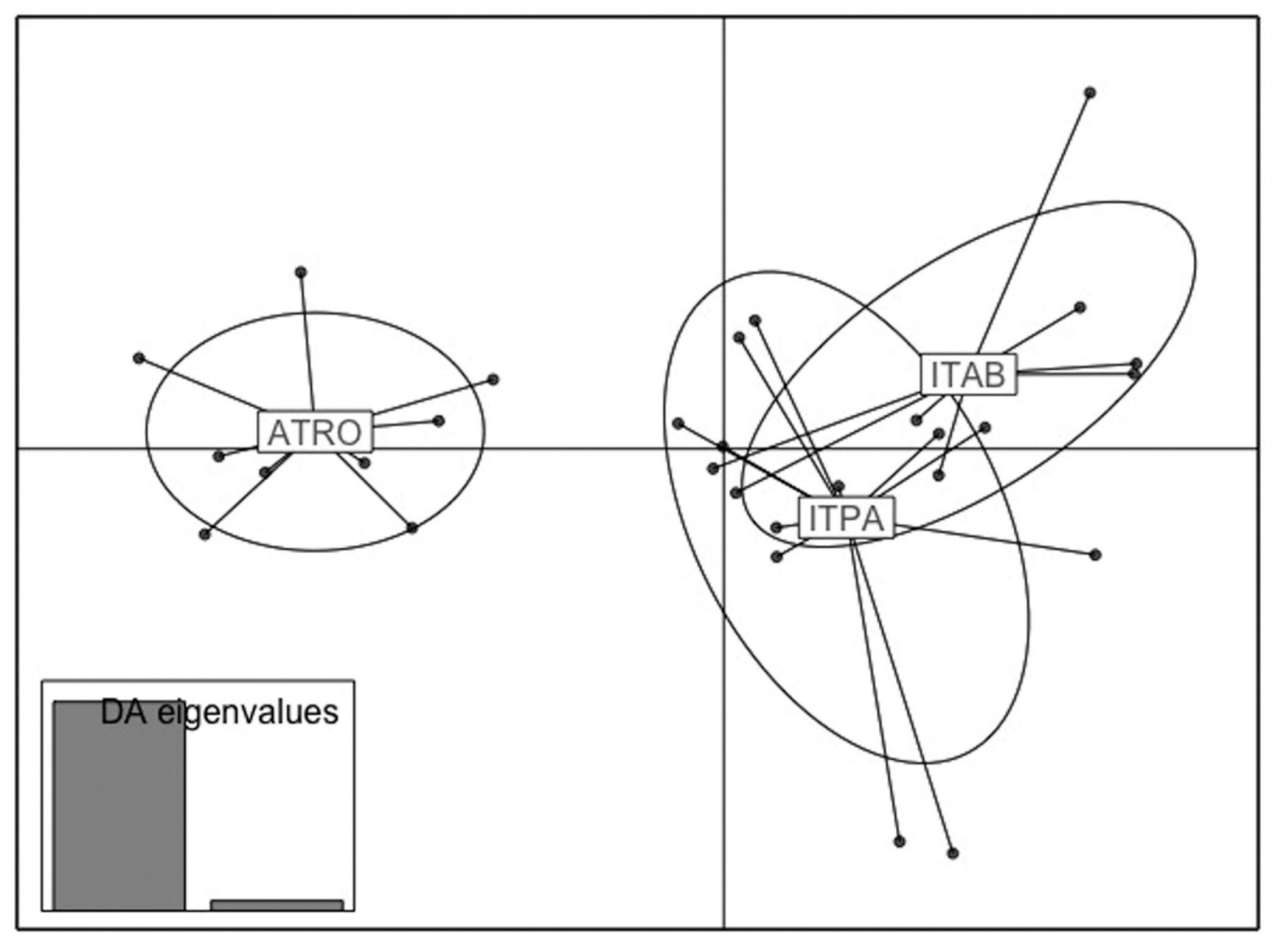
Population structure among *Pityogenes chalcographus* individuals, analysing a set of 5470 genome-wide markers applying a discriminant analysis of principal components (DAPC). Abbreviations: ATRO, Austria/Rothwald; ITAB, Italy/Abetone; ITPA, Italy/Pavullo.

The DAPC analysis on 5470 loci suggests a grouping of *P. chalcographus* individuals by geography. Individuals from the Austrian site, ATRO, were distinctly separated from samples from both Italian locations, ITAB and ITPA (Fig. 2). *Pityogenes chalcographus* from ITAB and ITPA, however, showed overlaps among each other. Furthermore, DAPC axes harbour information on the genetic structure across geography in fine resolution. The geographical distance between ATRO and ITPA is smaller than between ATRO and ITAB, as reflected by the genetic pattern in Figure 2.

The mean Nei’s pairwise *F*_ST_ value among all geographical sites was 0.034. The highest value was found between the Austrian site, ATRO, and the Italian location ITAB (= 0.040; for details, see Supporting Information, Table S3).

## Discussion

We performed a comparative population genetic study on the bark beetle *P. chalcographus*. Our results show that this species is structured in six *COI* clusters (clades/subclades) with differences in their spatial distribution across locations. Furthermore, it is noteworthy that a certain clade, PcIIIc, is private for the Apennines, supporting the hypothesis that this mountain range served as a glacial refugium. Analysis of a set of genome-wide SNPs revealed a distinct genetic structure across geography, with Apennine *P. chalcographus* clearly separated from Central European individuals. Taken together, our results support the hypothesis that this bark beetle survived Pleistocene ice ages in the Apennine Mountains, followed by postglacial range expansion, for example, to Central Europe.

### Population structure from analysis of mitochondrial data

Two studies analysing a part of the mitochondrial *COI* gene assessed the genetic structure of *P. chalcographus* (Avtzis *et al.*, 2008; Bertheau *et al.*, 2013). The relationship among mitochondrial haplotypes found in our study resembles the topology of phylogenetic trees reported by Avtzis *et al.* (2008) and Bertheau *et al.* (2013). All clades, except for PcIIId, were well supported, confirming previous findings (Avtzis *et al.*, 2008; Bertheau *et al.*, 2013).

However, differences in the proportions of mitochondrial clades/subclades per location were detected. In ATRO and ATPR, PcIIIa prevails, whereas other clades (PcI, PcII, PcIIIb and PcIIId) were found at lower frequencies. The results from ATRO resemble previous findings (Bertheau *et al.*, 2013), whereas stronger differences between our data from ATPR and those from Avtzis *et al.* (2008) were found. Avtzis *et al.* (2008) reported only PcI and PcIIIa from this location; the presence of PcII, PcIIIb and PcIIId was not reported previously. This might be because of a different sample size, with ten individuals analysed by Avtzis *et al.* (2008) and 47 in the present study.

In contrast to the Central European sites, PcI and PcIIIa were rarely found in both Apennine locations, whereas haplotypes assigned to PcIIIb and PcIIIc were prevalent. The distribution of clades in ITAB is relatively similar to the findings of Bertheau *et al.* (2013). Again, bigger differences of proportions of clades between previous work (Avtzis *et al.*, 2008) and our data were found in ITPA. In contrast to our study, samples belonging PcI and PcIIId were not found by Avtzis *et al.* (2008), which might also be explained by a slightly different sample size.

Overall, the distribution of mitochondrial clades in Central European and Apennine locations roughly confirms previous findings (Avtzis *et al.*, 2008; Bertheau *et al.*, 2013); however, our results from a more thorough sampling provide detailed insights into the genetic structure of *P. chalcographus*. Taken together, our data clearly show that the Apennine Mountains were an important region for the mitochondrial diversity of *P. chalcographus*.

### Population structure from analysis of genomic data

Use of a large number of genome-wide markers provides additional information on the biogeographical structure of *P. chalcographus* at high resolution. The Apennine and Austrian locations show a distinct clustering of individuals by geography. Individuals of both Apennine sites have a strong overlap among each other and are not clearly separated. The linear geographical distance between ITAB and ITPA is ~50 km, and *P. chalcographus* can disperse up to 80 km, aided by wind (Nilssen, 1984), easily facilitating the exchange of genetic material among these sites. Beetles from the Austrian and Italian locations cluster by geography, which could be the result of different Eurasian refugia during Pleistocene ice ages (Avtzis *et al.*, 2008; Bertheau *et al.*, 2013; Schebeck *et al.*, 2018a).

### Evolutionary history of *P. chalcographus*

*Pityogenes chalcographus* is a common bark beetle in the Palaearctic, with a complex life history. For instance, it is oligophagous on various conifer species, is polygynous, has a high fecundity and can produce up to three generations per year (Schwerdtfeger, 1929; Postner, 1974). Moreover, this bark beetle has a relatively long evolutionary history, because the most recent common ancestor of mitochondrial haplotypes has been estimated to have diverged ~100 000 years ago (Bertheau *et al.*, 2013). Several evolutionary drivers have been hypothesized to result in genetic population structure. Bertheau *et al.* (2009) found that Norway spruce is the main host tree of *P. chalcographus* and is favoured over other conifer species. However, host plant usage does not result in genetic differentiation in this bark beetle (Bertheau *et al.*, 2012). Future studies might focus on a potential lineage diversification of individuals among additional Pinaceae species, including, for example, different pine species and Norway spruce varieties in the range of *P. chalcographus* (Schmidt-Vogt, 1977; Tollefsrud *et al.*, 2015), using genomic techniques.

Furthermore, infections with heritable bacterial endosymbionts, which could alter the reproductive outcome of *P. chalcographus*, were hypothesized as evolutionary drivers, resulting in population structure. Although *Wolbachia* and *Spiroplasma* were detected in this bark beetle (Arthofer *et al.*, 2009; Schebeck *et al.*, 2018b), the infection frequency and the geographical infection pattern in Europe do not suggest an endosymbiont-driven biogeographical signal.

Current knowledge on the evolutionary history of *P. chalcographus* suggests that climatic fluctuations, such as Pleistocene glacial–interglacial cycling, were most likely to be the main drivers of population structure in this beetle (Avtzis *et al.*, 2008; Bertheau *et al.*, 2013; Schebeck *et al.*, 2018a). Range contractions during cold periods to multiple geographically isolated glacial refugia caused limited exchange of genetic material among populations, resulting in the phylogeographical pattern that we observe today. Such climate-driven range changes during glacial–interglacial cycling have been found to affect the distribution and genetic population structure of numerous organisms, thus playing a crucial role as evolutionary drivers (Taberlet *et al.*, 1998; Hewitt, 2004; Schmitt, 2007; Martinet *et al.*, 2018).

One major *P. chalcographus* glacial refugium was located in the Italian–Dinaric region; other refugia were described for the Russian plain and the Carpathian Mountains (Avtzis *et al.*, 2008; Bertheau *et al.*, 2013; Schebeck *et al.*, 2018a). An important feature of a glacial refugium is the presence of unique or private haplotypes (Hewitt, 1996). Previous studies using mitochondrial data suggested that the Apennines might have been a Pleistocene refugium for *P. chalcographus* (Avtzis *et al.*, 2008; Bertheau *et al.*, 2013). Our data reporting the presence of the private mitochondrial clade PcIIIc for the Apennine locations strongly support the hypothesis that this region played a significant role as refugium, probably a cryptic one within the major Italian–Dinaric area.

The Apennines were a Pleistocene refugium for many animal and plant species, thus being an important region for the distribution and population structure of, for example, European insects, mammals and vascular plants. This biogeographical pattern is also imprinted in their genetic architecture, as reflected, for example, by the presence of private haplotypes (Hewitt, 1996, 2000; Petit *et al.*, 2002; Hewitt, 2004; Schmitt, 2007; Ruedi *et al.*, 2008; Grassi *et al.*, 2009; Pilot *et al.*, 2010; Bogdanowicz *et al.*, 2015).

Another characteristic feature found in several bark beetle species is that they shared their glacial refugia with their main host trees (Cognato *et al.*, 2003; Avtzis *et al.*, 2008; Bertheau *et al.*, 2013). One Norway spruce refugium was suggested for the Apennine Mountains (Schmidt-Vogt, 1977; Giannini *et al.*, 1991), thus corroborating a *P. chalcographus* refugium in this region.

Furthermore, the phylogeographical signal of *P. chalcographus* is characterized by low levels of population differentiation (e.g. genetic distances among haplotypes, *F*_ST_ values). This is underlined by the presence of various mitochondrial haplotypes per population and overlaps of individuals among locations in the DAPC analysis. This pattern was also found in other bark beetle species, such as the European spruce bark beetle *Ips typographus* (Linnaeus, 1758) (Stauffer *et al.*, 1999; Bertheau *et al.*, 2013; Mayer *et al.*, 2015). Given that outbreeding bark beetles, such as *P. chalcographus* and *I. typographus* (Mayer *et al.*, 2015), disperse over long distances, individuals from different origins might exchange genetic material, reflected in this biogeographical pattern.

In conclusion, we showed the importance of the Apennine Mountains for the genetic structure of European *P.chalcographus*. We found a strong indication (the presence of a certain private mitochondrial clade) that this mountain range was a refugium during glacial periods. Moreover, *P. chalcographus* from Italy and Austria clearly clusters by geography, with overlaps of individuals among geographically close locations. Together with the presence of multiple mitochondrial clades per site, this suggests that beetles of different origins contributed to the genetic structure we observe today.

## Supplementary Material

Additional Supporting Information may be found in the online version of this article at the publisher's web-site:

Table S2

SI1, Table S1, Table S2, Table S3

## Figures and Tables

**Table 1:** Overview of *Pityogenes chalcographus* sample sites and sample sizes

Site	Abbreviation	Coordinates	*N (COI)*	*N* (ddRADSeq)
Austria/Prinzersdorf	ATPR	48°13′N, 15°31′E	47	–
Austria/Rothwald	ATRO	47°45′N, 15°04′E	47	9
Italy/Abetone	ITAB	44°08′N, 10°39′E	48	9
Italy/Pavullo	ITPA	44°20′N, 10°50′E	48	12

Abbreviations: *N (COI)*, sample size for analysis of the mitochondrial *COI* gene; *N* (ddRADSeq), sample size for genome-wide analysis using double digest restriction site-associated DNA sequencing.

**Table 2:** Mean (±SE) Kimura two-parameter genetic distances among mitochondrial *COI* clades of *Pityogenes chalcographus*

	PcI	PcII	PcIIIa	PcIIIb	PcIIIc
PcI					
PcII	0.015 (±0.004)				
PcIIIa	0.017 (±0.004)	0.015 (±0.004)			
PcIIIb	0.018 (±0.004)	0.015 (±0.004)	0.012 (±0.003)		
PcIIIc	0.018 (±0.004)	0.012 (±0.003)	0.010 (±0.003)	0.011 (±0.003)	
PcIIId	0.017 (±0.004)	0.012 (±0.003)	0.011 (±0.003)	0.011 (±0.003)	0.008 (±0.002)

Clade terminology follows Avtzis *et al.* (2008) and Bertheau *et al.* (2013).
