# Supplementary material for: The Apennines as a cryptic Pleistocene refugium of the bark beetle Pityogenes chalcographus (Coleoptera: Curculionidae)
Source: Biol J Linn Soc Lond. Author manuscript; Available in PMC 2019 Jun 10. (PMC6557710; doi:10.1093/biolinnean/blz012)
Supplement: SI1, Table S1, Table S2, Table S3 [file EMS83174-supplement-SI1__Table_S1__Table_S2__Table_S3.docx]

**Supplementary Information**

**Title:** The Apennines as cryptic Pleistocene refugium of the bark beetle *Pityogenes chalcographus* (Coleoptera: Curculionidae)

**Authors:** Martin Schebeck, Hannes Schuler, Birgit Einramhof, Dimitrios N. Avtzis, Eddy J. Dowle, Massimo Faccoli, Andrea Battisti, Gregory J. Ragland, Christian Stauffer, Coralie Bertheau

**Supplementary Information SI 1.**

The structure-file (P.chalcographus_Apennine.str) contains the genotype calls of in total 30 samples from 5,470 SNP loci: ATRO1-ATRO9 (ATRO = Austria/Rothwald), ITPA1-ITPA12 (ITPA = Italy/Pavullo), ITAB1-ITAB9 (ITAB = Italy/Abetone).

Table S1. Number of individuals and number of COI haplotypes per mitochondrial clade of *Pityogenes chalcographus*. ITAB = Italy/Abetone, ITPA = Italy/Pavullo, ATPR = Austria/Prinzersdorf, ATRO = Austria/Rothwald. Clade terminology after (Avtzis, Arthofer & Stauffer, 2008) and (Bertheau, Schuler, Arthofer, Avtzis, Mayer, Krumbock, Moodley & Stauffer, 2013).

|  |  | PcI | PcII | PcIIIa | PcIIIb | PcIIIc | PcIIId |
| --- | --- | --- | --- | --- | --- | --- | --- |
| ITAB | number of individuals | 0 | 7 | 4 | 13 | 14 | 10 |
|  | number of haplotypes | 0 | 6 | 3 | 6 | 3 | 3 |
| ITPA | number of individuals | 2 | 7 | 1 | 15 | 13 | 10 |
|  | number of haplotypes | 2 | 6 | 1 | 6 | 4 | 6 |
| ATPR | number of individuals | 5 | 1 | 30 | 3 | 0 | 8 |
|  | number of haplotypes | 5 | 1 | 17 | 2 | 0 | 7 |
| ATRO | number of individuals | 13 | 0 | 24 | 0 | 0 | 10 |
|  | number of haplotypes | 4 | 0 | 13 | 0 | 0 | 10 |

**Table S2 (at the end of this file).**

Table S2 shows pairwise genetic distances (Kimura-2-Parameter, i.e., K2P; with standard error) among mitochondrial haplotypes of *Pityogenes chalcographus*. Clade terminology follows (Avtzis et al., 2008) and (Bertheau et al., 2013)

Table S3. Pairwise F_ST_ values among geographic sites (ATRO = Austria/Rothwald, ITAB = Italy/Abetone, ITPA = Italy/Pavullo) analysing 5,470 genome-wide loci.

|  | ATRO | ITAB |
| --- | --- | --- |
| ATRO |  |  |
| ITAB | 0.040 |  |
| ITPA | 0.032 | 0.030 |

**References**

**Avtzis DN, Arthofer W, Stauffer C. 2008.** Sympatric occurrence of diverged mtDNA lineages of *Pityogenes chalcographus* (Coleoptera, Scolytinae) in Europe. *Biological Journal of the Linnean Society* **94:** 331-340.

**Bertheau C, Schuler H, Arthofer W, Avtzis DN, Mayer F, Krumbock S, Moodley Y, Stauffer C. 2013.** Divergent evolutionary histories of two sympatric spruce bark beetle species. *Molecular Ecology* **22:** 3318-3332.
